# Chronic Use of Proton Pump Inhibitors: A Potential Link to Amino Acid Deficiency and the Development of Depression

**DOI:** 10.7759/cureus.51067

**Published:** 2023-12-25

**Authors:** Jeevan J Murthy, Sarah Hughes, Colin Travis, Ankit Chalia, Samira Khan, Michael Ang-Rabanes, Raja Mogallapu

**Affiliations:** 1 College of Medicine, Eastern Division, West Virginia University School of Medicine, Martinsburg, USA; 2 College of Medicine, Eastern Division, West Virginia School of Osteopathic Medicine, Martinsburg, USA; 3 Department of Behavioral Medicine and Psychiatry, West Virginia University School of Medicine, Martinsburg, USA

**Keywords:** protein malabsorption, neurotransmitter, proton-pump inhibitors, gastroesophageal reflux disorder, major depressive disorder, gut-brain axis

## Abstract

In recent years, the gut-brain axis (GBA) has been implicated in several vital physiological processes, including digestion, immunity, inflammation, and mood regulation. Disruption of this network is tied to the development of several pathological conditions, including mood disorders, inflammatory bowel diseases, and dementia. Proton pump inhibitors (PPI) are among the most utilized and easily accessible medications worldwide. Although they are effective at treating conditions, including gastroesophageal reflux disorder (GERD), peptic ulcer disease, Zollinger-Ellison syndrome, and erosive esophagitis, PPIs have several mechanisms that may precipitate protein and, thus, amino acid malnutrition. Our patient is a 34-year-old female with a longstanding history of GERD treated with proton-pump inhibitors who presented to the psychiatry clinic complaining of a six-month history of depression without extraneous psychosocial factors. Although the patient refused psychiatric intervention, she desired an answer for her symptoms, leading to the discovery of a severe tyrosine deficiency. As tyrosine is critical in the process of neurotransmitter synthesis, replenishment of the amino acid along with discontinuation of proton-pump inhibitors was found to relieve her depressive symptoms within a few short months. In this report, we seek to establish a link between the chronic use of proton-pump inhibitor medications and the development of mood disorders.

## Introduction

Major Depressive Disorder (MDD) is the most commonly diagnosed mental disorder in the United States, believed to affect 1 in 10 Americans. Diagnosis requires at least two weeks of depressed mood alongside symptoms, including sleep dysfunction, anhedonia, guilt or worthlessness, poor concentration, alterations in baseline appetite, and suicidality. The highly postulated pathophysiology of MDD includes a lack of serotonin, which influences mood, and decreased dopamine and epinephrine, which contribute to motivation, energy levels, and pleasure activities. Standard of care employs a combination of cognitive behavior therapy and pharmacotherapy, including selective serotonin reuptake inhibitors (SSRI), serotonin and norepinephrine-reuptake inhibitors (SNRI), tricyclic antidepressants (TCA), monoamine-oxidase inhibitors (MAOIs), and many other drugs that do not fall into a particular class [1].

**Figure 1:**
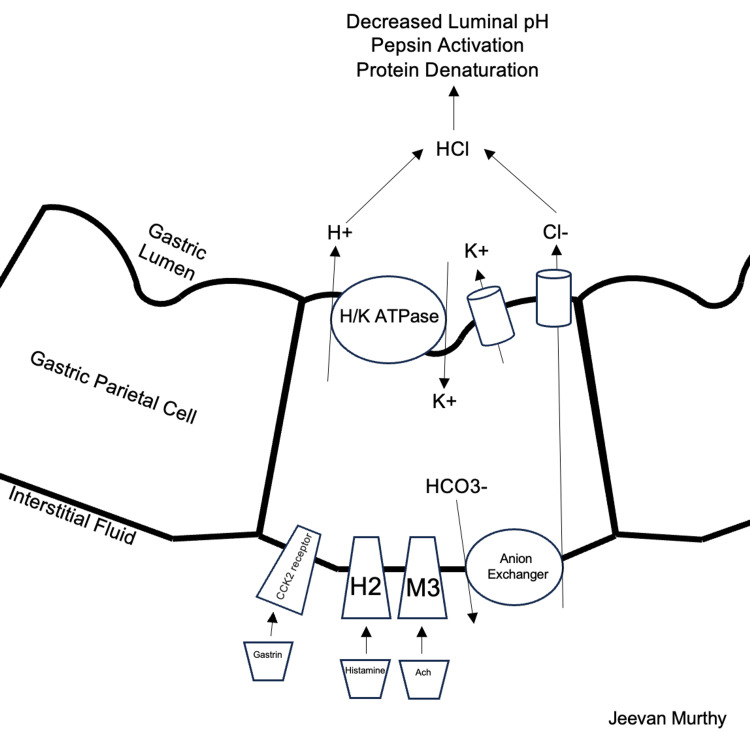
Process of hydrochloric acid production and secretion from gastric parietal cells. Image credit: Jeevan Murthy, West Virginia University School of Medicine

In recent years, more research has established the connection and interplay between the gastrointestinal tract, enteric nervous system (ENS), and central nervous system (CNS). This relationship, more formally known as the gut-brain axis (GBA), is a bidirectional communication system that plays several roles in physical and mental health processes (Figure 1). The GBA utilizes neurotransmitters to maintain homeostasis and comprises anatomical structures, including the vagus nerve, enteric nerve networks, and gut microflora. The ENS is known to sense direct alterations to the gut environment, including mucosal inflammation, disrupted microflora composition, and toxins, which promote vagal stimulation and an appropriate central stress response by the hypothalamic-pituitary-adrenal axis. Dysfunction of the GBA has been implicated in numerous pathological conditions, including mood disorders, inflammatory bowel disease, and neurodegeneration [2]. The specific relationships between GBA dysregulation and mood disorders, specifically major depressive disorder, are yet to be fully elucidated.

In this report, we seek to outline several mechanisms supporting our hypothesis that chronic use of proton-pump inhibitor medication may predispose patients to amino acid deficiency and subsequent development of major depressive disorder. We intend to frame this hypothesis using the case of a patient who presented with typical signs and symptoms of major depressive disorder but, upon further investigation, was revealed to have an easily treatable amino acid deficiency. Investigating underlying medical etiology may become more readily utilized in future psychiatric workups with this knowledge.

## Case presentation

Our patient is a 34-year-old female who presented to the psychiatry clinic concerning a six-month history of anhedonia, fatigue, weight gain, and general feelings of worthlessness. The symptoms became particularly distressing after they began impacting her occupation. Past medical history included three years of gastroesophageal reflux disorder requiring daily use of Pantoprazole 20 milligrams. She has never been diagnosed with any psychiatric condition and has no genetic predisposition. According to the patient, she consumed a well-rounded diet, avoided alcohol and tobacco use, and attempted to exercise regularly. She was in a healthy relationship and had two grown children. 

During the initial consultation, the patient scored twelve on the PHQ-9 screening, classifying her with moderate major depressive disorder. Further workups for reversible causes were negative for thyroid dysfunction (TSH, free T4), vitamin/mineral deficiency (iron, vitamin B12, vitamin D, magnesium), or autoimmune conditions (c-reactive protein, erythrocyte sedimentation rate, ANA levels). The patient desired an answer to her symptoms and was willing to finance extensive serum testing to identify a causal factor while simultaneously being unwilling to trial psychotropic medication. This included fatty acid and essential amino acid levels, which returned several weeks later. The patient was discovered to be severely deficient in the amino acid tyrosine (22 umol/L, normal range 38-96 umol/L), a critical building block in the structure of neurotransmitters implicated in clinical depression, including norepinephrine, dopamine, and serotonin.

PPI cessation and 500 mg L-tyrosine twice per day dramatically reduced her PHQ-9 score to four after just two months. The patient did have residual sleep difficulties but reported a significant improvement in her anhedonia and fatigue throughout the day. Tyrosine levels were rechecked at six months, showing a level of 51 umol/L. She was also referred to a gastroenterologist for the management of GERD, who transitioned her to H2-antagonist therapy. Furthermore, the nutritionist recommended a high-protein diet with additional lifestyle changes in the context of her GERD. This case highlights a peculiar association between chronic proton pump inhibitor use and amino acid deficiency, a factor postulated to predispose patients to depression and other psychiatric conditions.

## Discussion

**Figure 2:**
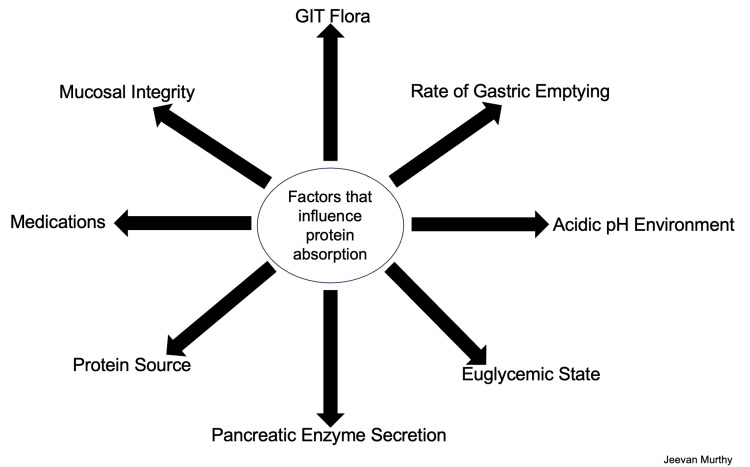
Factors that influence protein absorption in the gastrointestinal tract. Image credit: Jeevan Murthy, West Virginia University School of Medicine

Amino acids are the basic building blocks of proteins and are necessary components of a well-rounded diet. Protein catabolism begins in the upper gastrointestinal tract (GIT) with the release of hydrochloric acid (HCl) from parietal cells via the enzyme gastrin, which denatures the remaining large protein and polypeptide structures. HCl also activates pepsin, a digestive enzyme released by gastric chief cells that cleaves peptide bonds that are more readily absorbed in the GIT (Figure 1). In the proximal duodenum, the food mixture (now known as chyme) is further digested by pancreatic enzymes, including trypsins, chymotrypsins, and carboxypeptidases. The pancreas also secretes bicarbonate-rich juices that neutralize the acidic gastric products. Finally, brush-border enzymes found throughout the small intestine's duodenum, jejunum, and ileum cleave any remaining peptide bonds in the chyme [3]. Several factors impact protein absorption in the GIT, including a healthy mucosal lining, balanced gut flora, proper blood/lymphatic flow, and a robust immune system (Figure 2). Other factors like stable blood sugar levels, gastric emptying rate, and protein source type play a role [4]. However, one of the most critical factors for amino acid absorption is maintaining an adequate acidic environment in the stomach.

**Figure 3:**
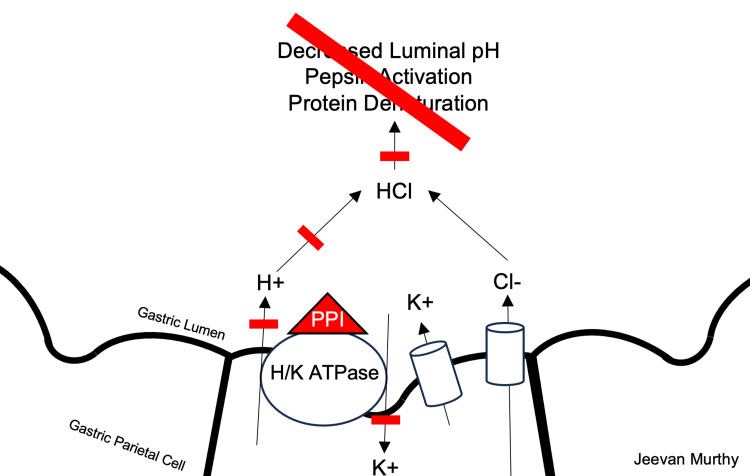
Mechanism of action of proton-pump inhibitor drugs. Image credit: Jeevan Murthy, West Virginia University School of Medicine

Proton pump inhibitors (PPI) are among the most commonly prescribed medications worldwide; some studies suggest that nearly 1/4 of adults utilize these medications daily [5]. PPIs are the mainstays of treatment for several gastrointestinal disorders, including gastrointestinal reflux disease (GERD), peptic ulcer disease, Zollinger-Ellison syndrome, and erosive esophagitis. For the treatment of GERD, the recommended time frame for PPI therapy is 4-8 weeks [6]. Mechanistically, PPIs target the final step in the stomach's production of hydrochloric acid. The active ingredients in PPIs form irreversible covalent bonds with the hydrogen-potassium ATPase enzyme (synonymous with "proton pump") located on the luminal surface of the gastric parietal cell, rendering them inactive in the secretory transport of hydrogen ions (Figure 3). Due to their irreversible nature, PPIs can exert their activity for several days at a time [7].

Long-term PPI use is associated with several non-specific symptoms, including GI upset, headache, and fatigue. Of more concern, however, is the increased risk of fractures, nutrient deficiencies (vitamin B12, vitamin D, magnesium), protein malnutrition, and infections, including *Helicobacter pylori* and *Clostridium difficile*, and rebound acid-hypersecretion with rapid cessation. Prolonged inhibition of the parietal cell stimulates increased gastrin secretion, promoting cell hypertrophy and reactionary secretion with drug cessation [8]. Protein and micronutrient malnutrition are especially concerning, as amino acids, electrolytes, and vitamins are critical substrates in numerous biological functions [9]. While the "normal" resting gastric pH is considered to be less than a pH of 3.0, longstanding PPI usage places the gastric environment at a pH of 5-7. In addition to impaired denaturation of proteins, activation of pepsin (from pepsinogen) is most significant at pH 2.0, while its peak protease activity is between 1.8 and 2.3 [10].

**Figure 4:**
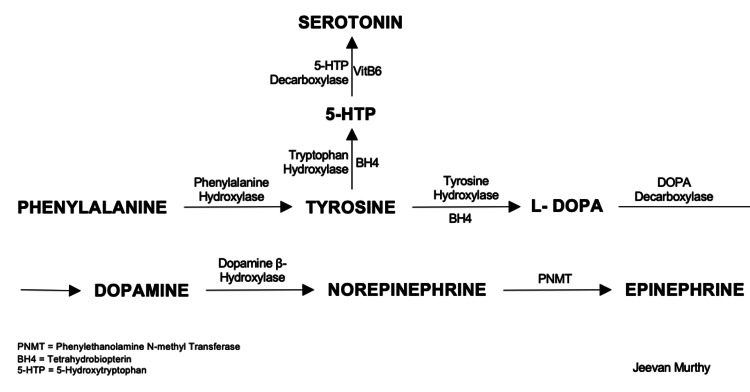
Neurotransmitter synthesis pathway, including enzymes, intermediates, and key cofactors. Image credit: Jeevan Murthy, West Virginia University School of Medicine

As mentioned earlier, amino acids are essential precursors to several biological functions, including synthesizing neurotransmitters. Although some interconversion is possible, several amino acids cannot be synthesized in humans and are essential components of food and supplementary nutrition. These include histidine, isoleucine, leucine, lysine, methionine, phenylalanine, threonine, tryptophan, and valine. Tyrosine is not included in this list; however, some studies have indicated that as people age, there may be limitations in total tyrosine synthesis [11]. Several steps in these pathways are outlined in the diagram below, including the way in which tyrosine can be converted to dopamine (Figure 4).

There are several proposed pathways and mechanisms by which proton-pump inhibitor medication may play a role in the synthesis of neurotransmitters. These include, but are not limited to, alterations to gastric pH, gut microflora disruption, and interference with critical enzymes and their cofactors. By examining these mechanisms, we intend to provide an understanding of the current literature and future directions of in vitro and in vivo studies. 

PPI drugs may have an ulterior mechanism as inhibitors of the enzyme tyrosine hydroxylase, which is involved in the synthesis of several catecholamines implicated in MDD and other psychiatric disorders. Betari and colleagues utilized in-vivo mouse models to demonstrate Omeprazole as a selective inhibitor of tryptophan hydroxylase (TPH1/TPH2), which has significant conservation in the active site of the other aromatic amino acid hydroxylases, namely tyrosine and phenylalanine hydroxylases. However, this same study found that the S-enantiomer of Omeprazole and Esomeprazole can also inhibit monoamine oxidase (MAO-A), an enzyme implicated in the metabolism of neurotransmitters, including 5HT, NE, and DA. They concluded that the MAO-A inhibition by high-dose Omeprazole overshadowed the opposing effect on 5HT produced by inhibition of TPH1 and TPH2 [12]. Despite the current literature, most of these pathways are relatively unknown.

While PPIs increase gastric pH and induce hypochlorhydria, a healthy subject taking PPIs should not have disruption of the gastric bactericidal barrier. Chronic use of PPIs can lead to small intestinal dysbiosis. One alteration is an overgrowth of *Streptococcaceae* and, subsequently, a replacement of the native gut bacteria. Several species driven out of the gut include the Lactobacillus and Bacillus families, which contribute to neurotransmitter production, potentially contributing to anxiety and depression [13]. Decreased acidity in the stomach can allow for altered bacterial flora to cross into the small intestine. This phenomenon is typically appreciated in the post-infection state, but less acutely, it may lead to other problems such as immune function impairment, adverse effects on metabolism, associations with neurocognitive disorders, and impaired nutrient absorption [14].

**Figure 5:**
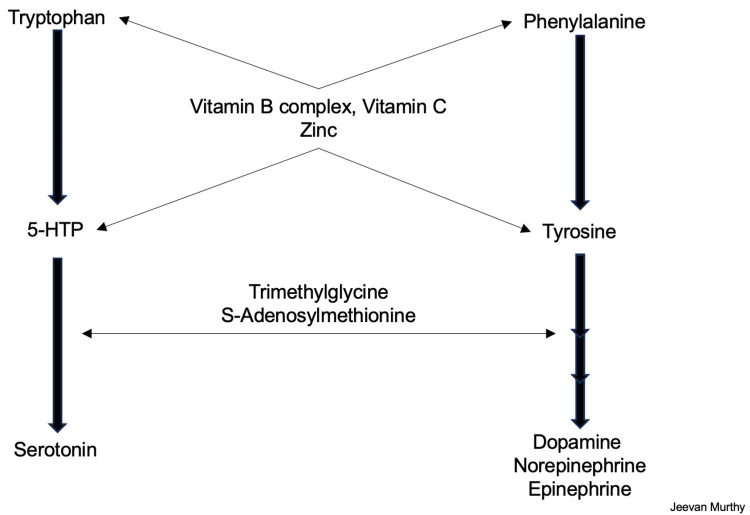
Minerals and other cofactors are implicated in the conversion of key neurotransmitters. Image credit: Jeevan Murthy, West Virginia University School of Medicine

Proton pump inhibitors may also limit catecholamine synthesis via the malabsorption of key enzymatic cofactors (Figure 5). In numerous studies, chronic PPI usage has been linked to vitamin B12 deficiency. Vitamin B12 is implicated in several pathways involving catecholamine synthesis, most notably as the major cofactor required for the activity of dopamine hydroxylase, the enzyme controlling the conversion of dopamine into norepinephrine [15]. Multiple meta-analyses have indicated that PPIs may also increase the incidence of hypomagnesemia. Magnesium modulates dopamine production by altering the expression of genes involved in dopamine production [16]. PPI-associated iron-deficiency anemia has also been observed; mechanisms for this have been posited, including upregulation of hepcidin, which blocks duodenal ferroportin, limiting iron absorption [14]. Iron is a known cofactor for tyrosine hydroxylase [17].

Several studies have explored the direct relationship between chronic PPI use and major depressive disorders. One recent study illustrated elevated anxiety, impaired learning and memory, and decreases in 5HT-1a receptor expression as well as serotonin levels in rats treated with either a medium (10 mg) or high (20 mg) dose of Omeprazole. The high dose of Omeprazole reduced 5HT-1A expression in the raphe nuclei and hippocampus, illustrating a pathophysiologic contribution to the increased risk of depression and anxiety [18]. Laudisio et al. assessed geriatric patients using the Geriatric Depression Scale (GDS) who were also taking PPIs. PPI use was not only associated with a high GDS score (p=0.014) but also a higher probability of clinical depression, defined as GDS ≥ 11 (p=0.045) [19]. This association was later explored with children. A prospective cohort study in Sweden assessed 58,640 children between 7-17 years old, including both initiators and non-initiators of PPIs. The primary outcome was an incident of anxiety and depression determined by either a formal diagnosis or the initiation of an SSRI. Their results indicated that PPI users had a 2.6-fold increased risk of developing anxiety and depression, with an increased probability of prolonged PPI use (p<0.0001) [20].

## Conclusions

In the future, more research is required to fully elucidate any potential links between PPI use and mood disorders, including anxiety and depression. Several areas of investigation include deeper analysis of the aforementioned biochemical pathways, improved understanding of patient-specific risk factors, better pharmacogenomics understanding of the drugs themselves, standardization of serum monitoring, and long-term cohort-based studies on PPI therapy. These goals will hopefully guide healthcare providers in delivering more personalized and effective care. It is also essential to consider the practicalities of such meticulous patient monitoring; many providers and patients may need more resources and access to individualized care than our patients did. Currently, routine monitoring of amino acid levels is expensive, time-insensitive, and an inefficient use of diagnostic tests. While the current evidence suggests a connection between PPI use and mood disorders, continued research is necessary to refine treatment approaches, identify at-risk populations, and develop more practical monitoring methods. 

In this report, we sought to establish a link between the chronic use of proton-pump inhibitor medications and the development of mood disorders, specifically manifested as major depressive disorder in our patient example. An integrated approach between psychiatry, gastroenterology, and dietary specialists promoted the rapid healing of this patient. Chronic PPI use may contribute to the development of mood disorders by disrupting amino acid metabolism and neurotransmitter anabolism through several mechanisms, including blockade of specific enzymes, dysregulation of gut microflora, malnutrition of vital nutritional cofactors, and, most importantly, prevention of adequate amino acid utilization through absorption of dietary proteins. Like many other medical comorbidities, including thyroid dysfunction, autoimmune conditions, and metabolic disorders, amino acid deficiency may someday be considered a "reversible" cause of mood disorders. We hope that this report may stimulate future studies to delve into the connection between PPI use and mood disorders, which will serve to inform the clinical relevance of chronic use of PPIs in specific patient populations.
